# Hypertrophic pyloric stenosis masked by kidney failure in a male infant with a contiguous gene deletion syndrome at Xp22.31 involving the steroid sulfatase gene: case report

**DOI:** 10.1186/s13052-022-01218-5

**Published:** 2022-02-03

**Authors:** Ingrid Anne Mandy Schierz, Mario Giuffrè, Marcello Cimador, Maria Michela D’Alessandro, Gregorio Serra, Federico Favata, Vincenzo Antona, Ettore Piro, Giovanni Corsello

**Affiliations:** 1 Neonatal Intensive Care Unit, Department of Health Promotion, Mother-Child Care, Internal Medicine and Medical Specialties “G. D’Alessandro”, University Hospital “P. Giaccone”, Via Alfonso Giordano n. 3, 90127 Palermo, Italy; 2 Pediatric Surgery Unit, Department of Health Promotion, Mother-Child Care, Internal Medicine and Medical Specialties “G. D’Alessandro”, University Hospital “P. Giaccone”, Palermo, Italy; 3 Department of Pediatric Nephrology, Children’s Hospital “G. Di Cristina”, Palermo, Italy

**Keywords:** Case report, Xp22.3 nullisomy, Congenital anomalies of the kidney and urinary tract, Gastric outlet obstruction, Digestive system abnormalities

## Abstract

**Background:**

Contiguous gene deletion syndrome at Xp22.3 resulting in nullisomy in males or Turner syndrome patients typically encompasses the steroid sulfatase gene (*STS*) and contiguously located other genes expanding the phenotype. In large deletions, that encompass also the Kallmann syndrome 1 gene (*KAL1*), occasionally infantile hypertrophic pyloric stenosis (IHPS) and congenital anomalies of the kidney and urinary tract (CAKUT) have been reported.

**Patient presentation:**

We report on a male newborn with family history in maternal uncle of renal abnormalities and short stature still without ichthyosiform dermatosis. The baby presented CAKUT with kidney failure and progressive vomiting. Renal bicarbonate loss masked hypochloremic and hypokalemic metabolic alkalosis classically present in IHPS and delayed its diagnosis. Antropyloric ultrasound examination and cystourethrography were diagnostic. After Fredet-Ramstedt extramucosal pyloromyotomy feeding and growing was regular and he was discharged home. Comparative whole-genome hybridization detected a maternal inherited interstitial deletion of 1.56 Mb on Xp22.31(6,552,712_8,115,153) × 0 involving the *STS* gene, but not the *KAL1* gene*.*

**Conclusions:**

Aberrant cholesterol sulfate storage due to *STS* deletion as the underlying pathomechanism is not limited to oculocutaneous phenotypes but could also lead to co-occurrence of both IHPS and kidney abnormalities, as we report. Thus, although these two latter pathologies have a high incidence in the neonatal age, their simultaneous association in our patient is resembling not a chance but a real correlation expanding the clinical spectrum associated with Xp22.31 deletions.

## Background

Contiguous gene deletion syndrome at Xp22.3 resulting in nullisomy in males or Turner syndrome patients is characterized by the combination of one or more monogenic disorders and clinical findings as short stature (short stature homeobox gene, *SHOX*), chondrodysplasia punctata (arylsulfatase genes - *ARSD*, *ARSE*, *ARSF*), X-linked ichthyosis (arylsulfatase C or steroid sulfatase gene, *STS*), ocular albinism type I (*OA1*) and elements of X-linked neurodevelopmental disorders and Kallmann syndrome (*KAL1*; reduced hypothalamic and pituitary function with resulting hypogonadotropic hypogonadism and hypoplasia of the olfactory bulb) [1], whereas the term Rud’s syndrome should no longer be used [2]. FG syndrome 3 is also mapped to this region [3]. In large deletions, occasionally cardiac arrhythmia [4], periventricular nodular heterotopia [5], acute lymphoblastic leukemia [6], end-stage renal failure [7] and infantile hypertrophic pyloric stenosis (IHPS) [3, 4, 8–12] were also reported. The most critical region of deletion breakpoints, characterized by a low frequency of interspersed repeats and a low GC content [13], encompasses the *STS* gene (MIM*300747) resulting in microsomal enzyme deficiency with an incidence about 1:1500 in males [14]. This membrane-bound enzyme is ubiquitously expressed and hydrolyzes several 3-beta-hydroxysteroid sulfates, which serve as metabolic precursors for estrogens, androgens, and cholesterol [15]. Despite the widespread enzyme deficiency, patients apparently have abnormalities only of the stratum corneum where increased cholesterol sulphate concentrations are causing abnormal desquamation, decreased corneodesmosomal degradation and retention hyperkeratosis of the skin mostly a few weeks after birth, but conatal collodion is also reported [12]. There might be associated cardiac arrhythmia and benign Pre-Descemet corneal dystrophy characterized by cholesterol sulfate accumulation and punctiform opacities without vision impairment on the one side, as well as cryptorchidism and neurobehavioral disorders due to deficient (neuro-) steroids on the other side [4, 16]. Despite the escape of lyonization, some female deletion carriers also have corneal opacities and can present parturition disturbances and cervical dystocia due to lacking placental production of estriol [4, 14]. Congenital anomalies of the kidney and urinary tract (CAKUT) have been reported rarer in *STS* limited microdeletions or point mutations than in larger deletions of Xp22.3 that encompass also the *KAL1* gene, a neighboring gene important for urogenital development [7, 12, 13, 17, 18].

We report on a male newborn with family history in maternal uncle of renal abnormalities and short stature still without ichthyosiform dermatosis. The baby presented CAKUT with kidney failure and progressive vomiting. Renal bicarbonate loss masked hypochloremic and hypokalemic metabolic alkalosis classically present in IHPS and delayed its diagnosis. This report of associated *STS* deletion and IHPS further define and expand the clinical spectrum associated with CNV in this region and provide support for the role of modifiers contributing to phenotypic variability.

## Patient presentation

This male term newborn is the second son of healthy non consanguineous Caucasian parents. His maternal uncle suffering from nephropathy had undergone a kidney transplant. Fetal sonographic assessment revealed hydronephrosis bilaterally, and oligohydramnios inducted to Caesarean section. At birth baby’s weight was 2710 g (− 1.07 SDS/10th centile), length 46 cm (− 1.82 SDS/3rd centile), and head circumference 33 cm (− 1.09 SDS/14th centile). During the first week of life, he developed severe acidosis and was referred to our department. Physical examination was unremarkable except for pale skin and hyporeactive aspect; male genitals were normal. There were no edemas. Diuresis, and blood pressure were normal. Laboratory investigations diagnosed renal insufficiency by low bicarbonates 15 mmol/l, augmented creatininemia 3.12 mg/dl, urea 89 mg/dl, chlor 120 mEq/l, moderate proteinuria 327 mg/l, glucosuria 500 mg/l and microhematuria, while anion gap, albuminemia, proteinemia and uric acid were preserved. Abdominal ultrasound and subsequent voiding cystourethrography showed renal hypoplasia on the left and renal dysplasia on the right as well as moderate hydronephrosis due to grade IV vesicoureteral reflux. X-ray, cranial and cardiac ultrasounds and electrocardiogram were normal. He started intravenous rehydration and bicarbonate supplementation. Refeeding by breast milk and a special powdered feed with low levels of potassium for renal impairment (Kindergen® 1 g in 5 ml water) was initiated after 12 h. He tended to have regurgitations attributed to a urinary infection and treated on the fifth day of the hospital stay with oral amoxicillin switched to oral cefixime on day 14 (sensitive to *Escherichia coli*) until negative urinary cultures were reported. Persistent regurgitation did not ameliorate by trials of smaller, more frequent feeds, thickened formula, and anti-Trendelenburg positional management. At 1 month of age, intermittent nonbilious vomiting increased markedly, he weighed 3110 g (< 0.4 centile), creatininemia and urea were halved, bicarbonates kalium and chlor were normal.

**Fig. 1:**
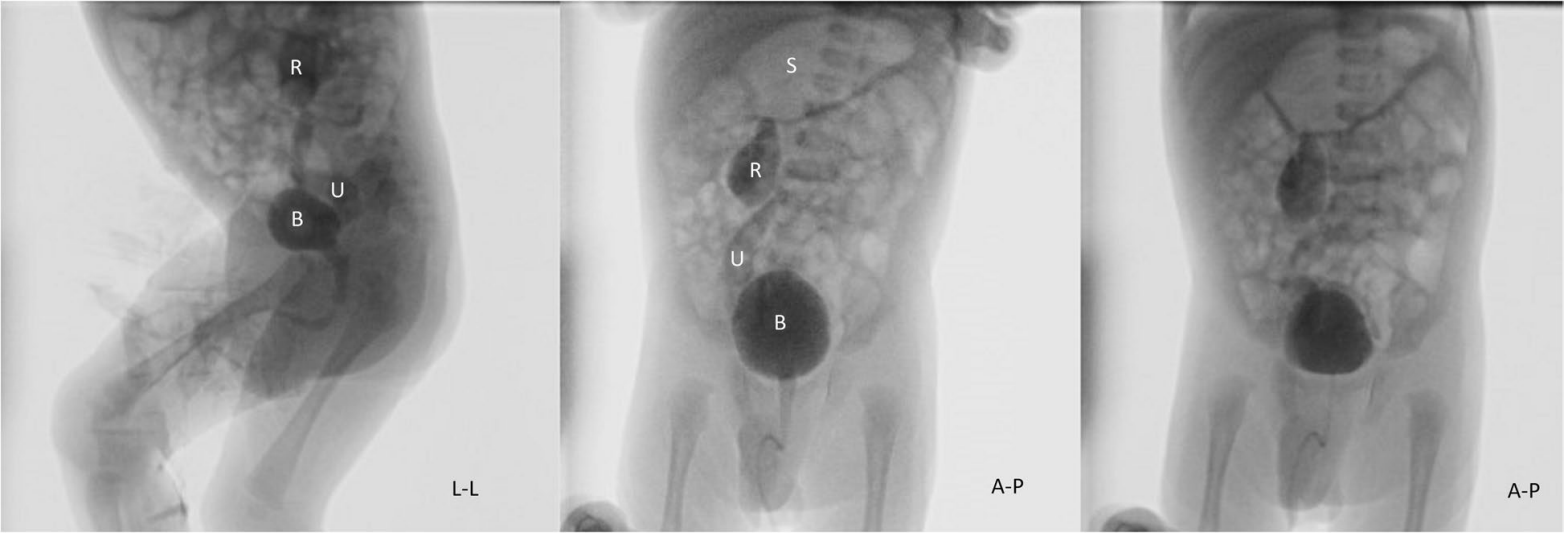
At 24 days of life, voiding cystourethrogram is showing absence of bladder (B) and urethral abnormalities, but right-sided vesicoureteral reflux with moderate dilatation of the ureter (U), renal pelvis (R) and calyces and blunting of fornices as well as accidental finding of persistent distended stomach (S) with undulating contours known as Caterpillar sign, virtually pathognomonic for hypertrophic pyloric stenosis

Antropyloric ultrasound examination revealed hypertrophied muscular layer of 4.6 mm and elongation of the pyloric canal of 19 mm (diameter 14 mm). In retrospect, some frame of the cystourethrogram had already shown an air-filled stomach with undulating contours known as “Caterpillar sign” (Fig. 1). Fredet-Ramstedt extramucosal longitudinal pyloromyotomy was performed. Afterwards, feeding and growing was regular and he was discharged home 45 days old. All treatment options have been discussed with both parents. Erythropoietin treatment and clinical multidisciplinary follow-up are ongoing. At 6 month of age, large polygonal, brownish scales appeared particularly on the anterior aspect of the lower extremities.

Comparative whole-genome hybridization was performed during hospitalization using the Agilent® 8x60K microarray and detected a maternal inherited interstitial deletion of 1.56 Mb on arr [hg19] Xp22.31(6,552,712_8,115,153) × 0 involving the genes *STS*, variable charge X-linked (*VCX*; *300229), pseudouridine 5′-phospatase (*PUDP*; *306480), Patatin like phospholipase domain containing-4 (*PNPLA4*; *300102) and microRNA MIR4767 and MIR651.

## Discussion and conclusion

We report on a male newborn with nonaccidental association of IHPS, kidney failure and maternal Xp22.3 deletion involving the *STS* gene.

IHPS is the most common form of gastrointestinal obstruction in infancy (1:700), five times more frequent in males than in females and hereditably is high as 87% [19, 20]. Isolated and syndromic IHPS are described [21]. The exact etiology of isolated IHPS is unknown, although neuronal nitric oxide synthase (NOS) upregulation and an extracellular matrix abnormality have been reported in subsets [21]. Various potential genetic loci have been investigated, as well as various environmental factors (maternal smoking or young age, firstborn, feeding practice, post-natal erythromycin use) without producing conclusive data. Interestingly, reducing erythromycin indications and increasing dietary intake of omega-3 fatty acids in Western countries during the last decade probably decreased the incidence of isolated IHPS [20, 22]. By studying syndromic IHPS (Table 1) it was evidenced that the lipid metabolism plays a fundamental role in etiopathogenesis [23]. The risk of IHPS is inversely and significantly associated with total cholesterol level with an Odds ratio of 0.77 (95% CI, 0.64–0.92; *p* = 0.005) per 10 mg/dL [24]. Indeed, there are higher incidence of IHPS in syndromes affecting the lipid metabolism. A classic example is the Smith-Lemli-Opitz syndrome, an autosomal recessive congenital disorder caused by mutations in the 7-dehydrocholesterol reductase (*DHCR7*) gene at 11q13. Affected individuals are unable to complete the final step in cholesterol biosynthesis with accumulation of aberrant 7-dehydrocholesterol in developing tissues causing a wide range of metabolic and developmental abnormalities, including IHPS in 10–15% of cases [21]. In congenital generalized lipodystrophy type IV (*CAVIN1* gene at 17q21) diffuse skeletal and smooth muscle hypertrophy are leading to cardiac arrythmia and IHPS [21, 25]. In syndromes associated with hypotonia, as in FG syndrome 3 (Xp22.3) or Down syndrome (critical region 21q22.3), the IHPS incidence is about 7% [3, 21]. Other syndromes frequently associated with IHPS are connective tissue disorders in which abnormal or excess of connective tissue in the pylorus gradually develop mechanic obstruction [21]. Furthermore, biopsies have shown not only muscle layer hypertrophy but also accumulation of extracellular matrix molecules (chondroitin-sulfate proteoglycan and fibronectin) [26]. This is also the underlying cause for unsuccessful non-surgical conservative treatment with oral or intravenous administration of atropine, leaving the surgical extramucosal pyloromyotomy as the gold standard [27].

**Table 1:** Differential diagnosis of syndromic infantile hypertrophic pyloric stenosis (IHPS) and renal anomalies as variable features (Bioinformatics were obtained from open-source GeneCards.org and MalaCards.org and from Peeters et al. [21])

Cytogenetic region/gene(s)	n. of IHPS cases	Mode of inheritance	Phenotype
1p36/*SKI, SPEN, RERE, PRDM16, GABRD, HSPG2*	1–4		1p36 deletion syndrome (craniofacial dysmorphism, hydrocephalus, genitourinary and neurodevelopmental disorders)
2q22.3/*ZEB2*	1–4	AD	Mowat-Wilson syndrome (craniofacial dysmorphism, deep set eyes, Hirschsprung disease, hydronephrosis)
2q37.3/*HDAC4*	1–4	AD	2q37 microdeletion syndrome (round face, multicystic kidneys, neurodevelopmental disorders)
3p25	1–4	AD	3p25 microdeletion syndrome (trigonocephaly, microcephaly, cardiac and genitourinary malformations, neurodevelopmental disorders); Noonan syndrome 5 (3p25.2/*RAF1* mutations)
4q22.1/*PKD2*	1–4	AD	Polycystic kidney disease 2, laterality defects
5p13.2/*NIPBL*	11–50	AD	Cornelia de Lange syndrome (microbrachicephaly, synorphrys, growth retardation, genitourinary malformations, cardiac and neurodevelopmental disorders)
6p12.3-p12.2/*PKHD1*	1–4	AR	Polycystic kidney disease 4, Caroli disease
6p24.3/*TFAP2A*	1–4	AD	Branchiooculofacial syndrome (orofacial clefts, hearing loss, renal agenesis or cystic anomalies)
6q15/*MAP 3 K7*	1–4	AD	Frontometaphyseal dysplasia 2, cardiac and genitourinary malformations
7q21.2/*PEX1*	1–4	AR	Zellweger syndrome (extreme hypotonia, seizures, renal and hepatic cysts/dysfunction)
8q12.2/*CHD7*	1–4	AD	CHARGE syndrome (coloboma, heart anomaly, choanal atresia, genitourinary and ear malformations); Kallmann syndrome (anosmia, hypogonadotropic hypogonadism)
10q24.32/*NFKB2*	1–4	AD	Common variable immunodeficiency-10, nephrotic syndrome
10q26/*FGFR2*	1–4	AD	Apert syndrome (craniosynostosis, complete syndactyly, hydronephrosis); Beare-Stevenson syndrome (craniosynostosis, cutis gyrate)
11p13/*WT1*	1–4	AD	Denys-Drash syndrome (genitourinary malformations and neoplasia)
11p15.5/*HRAS*	5–10	AD	Costello syndrome (fetal overgrowth, craniofacial dysmorphism, periorificial papillomata, echogenic kidneys, cardiomyopathy, neurodevelopmental disorders)
11q13.4/*DHCR7*	11–50	AR	Smith-Lemli-Opitz syndrome (short stature, craniofacial dysmorphism, cleft palate, genitourinary malformations, syndactyly of second and third toes, cardiac and neurodevelopmental disorders)
12q23.2/*PAH*	5–10	AR	Phenylketonuria (microcephaly, pale pigmentation, neurodevelopmental disorders if not recognized)
12q24.11/*UBE3B*	1–4	AR	Kaufman oculocerebrofacial syndrome (facial dysmorphism, cardiac, genitourinary malformations and neurodevelopmental disorders)
12q24.13/*PTPN11*	1–4	AD	Noonan syndrome 1 (short stature, facial dysmorphism, wolly hair, webbed neck, cardiac and genitourinary malformations)
Trisomy 13	1–4		Patau syndrome (hypotelorism, orofacial clefts, polydactyly, aplasia cutis, visceral malformations)
14q13.2/*PPP2R3C*	1–4	AR	Gonadal dysgenesis, dysmorphic facies, retinal dystrophy, myopathy
14q32	1–4	AD	Temple syndrome (short stature, maternal disomy)
16p13.3	1–4	AD	Polycystic kidney disease 1, intracranial aneurysm
16q22.2/*DHODH*	1–4	AR	Miller syndrome (postaxial acrofacial dysostosis, genitourinary malformations)
17q12/*HNF1B*	1–4	AD	HNF1B-related tubulointerstitial kidney disease, diabetes
17q21/*CAVIN1*	5–10	AR	Congenital generalized lipodystrophy type IV (muscular dystrophy, arrhythmia, phlebomegaly)
17q21.31/*KANSL1*	1–4	AD	Koolen-De Vries syndrome (craniofacial dysmorphism, cardiac and genitourinary malformations)
Trisomy 18 (18p)	5–10		Edwards’ syndrome (craniofacial dysmorphism, omphalocele, verticaltalus, visceral malformations)
18p11/*PIEZO2*	1–4	AD	Marden-Walker syndrome (microcephaly, blepharophimosis, arthrogryposis, genitourinary malformations)
18q21.32/*CCBE1*	1–4	AR	Hennekam lymphangiectasia-lymphedema syndrome
19q13.12/*NPHS1*	5–10	AR	Nephrotic syndrome type 1, hyperlipidemia
19p13.2/*ZNF699*	1–4	AR	DEGCAGS syndrome (neurodevelopmental disorders, visceral malformations)
19q13.2/*LTBP4*	1–4	AR	Cutis laxa type Ic (hydronephrosis, bladder diverticula)
20q13.33/*SOX18*	1–4	AD	Glomerulonephritis, hypotrichosis, lymphedema, telangiectasia
Trisomy 21	> 50		Down syndrome (hypotonia, craniofacial dysmorphism, sandal gap, cardiac and gastrointestinal malformations, neurodevelopmental disorders)
21q22.3/*COL18A1*	1–4	AR	Knobloch syndrome (eye and CNS abnormalities, aplasia cutis, duplex kidneys or ureters)
22q11.2/*BCR, MAPK1*	1–4		22q11.2 microdeletion syndrome
Xp11/*SMC1A*	11–50	XL	Cornelia de Lange syndrome (microbrachicephaly, synorphrys, genitourinary malformations, neurodevelopmental disorders)
Xp11.4/*BCOR*	1–4	XL	Lenz microphthalmia, genitourinary malformations
**Xp22/*STS, FGS3, KAL1***	**5–10**	**XL**	**X-linked ichthyosis; FG syndrome (hypotonia, macrocephaly, craniofacial dysmorphism, anorectal malformations); Kallmann syndrome (anosmia, hypogonadotropic hypogonadism)**
Xq11.2/*AMER1*	1–4	XL	Osteopathia striata, macrocephaly, cranial sclerosis, multicystic kidneys, male lethality
Xq13/*MED12*	5–10	XL	FG syndrome type 1 also known as Opitz-Kaveggia (hypotonia, macrocephaly, anorectal malformation)
Xq26.2/*GPC3*	1–4	XL	Overgrowth, organomegaly
Xq28/*FLNA, NAA10*	1–4	XL	Pseudoobstruction, hydronephrosis, aortic valvular dysplasia; Lenz microphthalmia; frontometaphyseal dysplasia

*Abbreviations*: *AD* Autosomal dominant, *AMER1* APC membrane recruitment protein 1, *AR* Autosomal recessive, *BCOR* corepressor for B-cell lymphoma 6, *BCR* Breakpoint cluster region, *CAVIN1* Caveolae associated protein 1, *CCBE1* Collagen and calcium-binding EGF domains 1, *CHD7* Chromodomain helicase DNA binding protein 7, *CNS* Central nervous system, *COL18A1* Collagen type XVIII alpha 1 chain, *DHCR7* 7-dehydrocholesterol reductase gene, *DHODH* Dihydroorotate dehydrogenase gene, *PKHD1* ciliary IPT domain containing fibrocystin/polyductin, *FGFR2* fibroblast growth factor receptor 2, *FGS3* FG syndrome 3, *FLNA* Filamin A, *GABRD* Gamma-aminobutyric acid type A receptor subunit delta, *GPC3* Glypican 3, *HDAC4* Histone deacetylase 4, *HNF1B* Hepatocyte nuclear factor-1-beta, *HRAS* HRas Proto-Oncogene, *HSPG2* Heparan sulfate proteoglycan 2, *KAL1* anosmin 1, *KANSL1* KAT8 regulatory NSL complex subunit 1, *LTBP4* Latent transforming growth factor beta binding protein 4, *MAP 3 K7* Mitogen-activated protein kinase kinase kinase 7, *MAPK1* Mitogen-activated protein kinase 1, *MED12* Mediator complex subunit 12, *NAA10* N-alpha-acetyltransferase 10 NatA catalytic subunit, *NFKB2* Nuclear factor kappa B subunit 2, *NIPBL* Nipped-B-like, *NPHS1* Nephrin, *PAH* Phenylalanine hydroxylase, *PEX1* Peroxisomal biogenesis factor 1, *PIEZO2* Piezo type mechanosensitive ion channel component 2, *PKD2* Polycystin, *PPP2R3C* Protein phosphatase 2 regulatory subunit B-double prime gamma, *PRDM16* PR/SET domain 16, *PTPN11* Protein tyrosine phosphatase non-receptor type 11, *RAF1* Raf-1 proto-oncogene, *RERE* Arginine-glutamic acid dipeptide repeats, *SKI* SKI proto-oncogene, *SMC1A* Structural maintenance of chromosomes 1A, *SOX18* SRY-box transcription factor 18, *SPEN* Spen family transcriptional repressor, *STS* Steroid sulfatase, *TFAP2A* transcription factor AP-2 alpha, *UBE3B* Ubiquitin protein ligase E3B, *WT1* Wilms tumor 1 transcription factor, XL X-linked, *ZEB2* Zinc finger E-box binding homeobox 2, *ZNF699* Zinc finger protein 699

*STS* alterations as in our case report, can lead to disturbed intracellular metabolism of cholesterol and to storage phenomenon of cholesterol sulphate. It was evidenced that age of onset of ichthyosis or absent/mild forms of XLI, frequently found in Southern European countries, are not related to width of Xp22.3 deletion [12, 18]. The late-onset of cutaneous presentation in our newborn is possible and clinical follow up have to direct dermatological, nephrological, endocrinological and neurobehavioral care as well as infection surveillance. *VCX*, *PUDP* and mitochondria-related *PNPLA4* have been implicated in neurocognitive development, although the functional significance of these genes remains under debate [11, 13]. *KAL1* gene, implicated in urogenital development, is not deleted in our case.

Vomiting and growth failure present a clinical challenge in neonatal age. Major causes are severe gastroesophageal reflux, neonatal sepsis, anatomical and functional gastrointestinal obstructions including IHPS and pylorospasm; less frequent are food allergy, inborn errors of metabolism, congenital adrenal hyperplasia, intracerebral abnormalities such as subdural hemorrhage or hydrocephalus, drugs or toxic agents and/or renal tubular acidosis. This spectrum widens in case of CAKUT, as in our patient, including renal impairment, risk of urosepsis and renal adapted diet. A concomitant edema could involve also the antropyloric region and cases of IHPS have been described [28]. Interestingly, a frequent recurrence linked polycystic kidney disease (PKD) and IHPS to NOS deficiency [29, 30]. Renal neuronal NOS and inducible NOS in cystic epithelium are suppressed or lost in PKD rats [31]. Thus, NOS deficiency leads to lack of locally available nitric oxide which may cause pyloric stenosis as a result of failure of smooth muscle relaxation. Downregulation of nitric oxide production may also be involved in the pathogenesis of pyloric stenosis in this subset. On the other hand, it was shown that deficiency of *STS* in kidneys results in increased cholesterol sulfate accumulation which interferes with normal functioning of transglutaminase 1, responsible for maintaining the integrity of cadherin-based adherens junctions between epithelial cells. The slit diaphragm of glomerular visceral epithelial cells is a modified adherens junction and, therefore, disruption of its structure by the above mechanism can result in proteinuria [7].

In conclusion, aberrant cholesterol sulfate storage due to *STS* deletion as the underlying pathomechanism is not limited to oculocutaneous phenotypes but could also lead to co-occurrence of both IHPS and kidney abnormalities, as we report. Thus, although these two latter pathologies have a high incidence in the neonatal age, their simultaneous association in our patient is resembling not a chance but a real correlation expanding the clinical spectrum associated with Xp22.31 deletions.

## Data Availability

The datasets used and/or analyzed during the current study are available from the corresponding author on reasonable request.

## Abbreviations

*ARSD*: Arylsulfatase gene D
CAKUT: Congenital anomalies of the kidney and urinary tract
*CAVIN1*: Caveolae associated protein 1
*DHCR7*: 7-dehydrocholesterol reductase
IHPS: Infantile hypertrophic pyloric stenosis
*KAL1*: Kallmann syndrome 1 gene
NOS: Nitric oxide synthase
*OA1*: Ocular albinism type I
PKD: Polycystic kidney disease
*PNPLA4*: Patatin like phospholipase domain containing-4
*PUDP*: Pseudouridine 5′-phospatase
*SHOX*: Short stature homeobox gene
*STS*: Steroid sulfatase gene
*VCX*: Variable charge X-linked
